# Currently Debated Topics on Surgical Treatment of Pancreatic Ductal Adenocarcinoma: A Narrative Review on Surgical Treatment of Borderline Resectable, Locally Advanced, and Synchronous or Metachronous Oligometastatic Tumor

**DOI:** 10.3390/jcm12206461

**Published:** 2023-10-11

**Authors:** Sergio Pedrazzoli

**Affiliations:** University of Padua, Via Crescini, 39, 35126 Padova, Italy; sergio.pedrazzoli@unipd.it; Tel.: +39-049-754386

**Keywords:** pancreatic cancer, borderline resectable, locally advanced, synchronous/metachronous oligometastatic, neoadjuvant chemo/radiotherapy, CA 19-9, ^18^F-FDG-PET

## Abstract

Background: Previously considered inoperable patients (borderline resectable, locally advanced, synchronous oligometastatic or metachronous pancreatic adenocarcinoma (PDAC)) are starting to become resectable thanks to advances in chemo/radiotherapy and the reduction in operative mortality. Methods: This narrative review presents a chosen literature selection, giving a picture of the current state of treatment of these patients. Results: Neoadjuvant therapy (NAT) is generally recognized as the treatment of choice before surgery. However, despite the increased efficacy, the best pathological response is still limited to 10.9–27.9% of patients. There are still limited data on the selection of possible NAT responders and how to diagnose non-responders early. Multidetector computed tomography has high sensitivity and low specificity in evaluating resectability after NAT, limiting the resection rate of resectable patients. Ca 19-9 and Positron emission tomography are giving promising results. The prediction of early recurrence after a radical resection of synchronous or metachronous metastatic PDAC, thus identifying patients with poor prognosis and saving them from a resection of little benefit, is still ongoing, although some promising data are available. Conclusion: In conclusion, high-level evidence demonstrating the benefit of the surgical treatment of such patients is still lacking and should not be performed outside of high-volume centers with interdisciplinary teams of surgeons and oncologists.

## 1. Introduction

According to recent epidemiological studies, pancreatic ductal adenocarcinoma (PDAC) is the 10th–14th most common cancer type and the 4th–7th leading cause of cancer death in the US and worldwide [1,2,3]. The incidence rate for PDAC has increased by about 1% per year since the late 1990s in both men and women [3,4]. PDAC is one of the dreaded malignancies for both the patient, as it is associated with a poor survival rate and decreased quality of life due to local invasion and complications, and the clinician, as it is challenging to diagnose [5]. In 2023, an estimated 64,050 new cases of pancreatic cancer will be diagnosed in the US, and 50,550 people will die from the disease [3,4]. For all stages combined, the 5-year relative survival rate is 12%, and even for the small percentage (15%) of people diagnosed with localized disease, the 5-year survival rate is only 44% [4]. According to Siegel et al. [3], the stage distribution for pancreatic cancer in the United States between 2015 and 2019 was as follows: 14–15% localized, 27–29% regional, 46–48% with distant metastases, and 10–1% unstaged. Unfortunately, at diagnosis, only 20% of patients are considered resectable and belong almost exclusively to the group with localized disease. A median relapse-free survival of 10–11.7 months [6,7] and a 5-year survival rate of 12–27% [8,9] are reported after potentially curative resection. A distinct subset of PDAC began to emerge in the early 2000s that blurred the distinction between resectable and locally advanced (LA), unresectable, disease: “marginally resectable” [10] or “borderline resectable” PDAC (BR-PDAC) [11]. Furthermore, until recently, metastatic PDAC, whether synchronous or metachronous, was considered unresectable regardless of the number, location, and resectability of metastases. However, the improvement and spreading of neoadjuvant chemotherapy allowed surgical resection to be performed in 30–40% of patients with borderline resectable and locally advanced PDAC [12]. Thanks to a greater effectiveness of oncological treatments and an increasing experience with surgical procedures, particularly with vascular resections [13,14], several patients previously considered unresectable have successfully undergone surgical resection.

This review will summarize the current evidence and the latest insights in the surgical management of PDAC regarding the main groups of patients who contributed to the enlargement of indications for surgery in the PDAC: (1) borderline resectable and locally advanced PDAC (BR-LA-PDAC), (2) synchronous oligometastatic PDAC, and (3) metachronous oligometastatic PDAC after potentially curative resection.

## 2. Borderline Resectable and Locally Advanced PDAC

### 2.1. Definition History

An R-0 resection is considered the main target of PDAC surgical treatment. An evaluation of the resectability of the PDAC is, therefore, the most important goal of the initial diagnostic procedures. A multidetector computed tomography (MDCT) scan usually allows PDAC to be classified as resectable (stage I or II), locally advanced (stage III), or metastatic (stage IV). However, thanks to advances in pancreatic imaging and surgical techniques, a distinct subset of cancers began to emerge in the early 2000s that blurred the distinction between resectable and locally advanced (LA) disease: “marginally resectable” [10] or “borderline resectable” PDAC (BR-PDAC) [11]. At that time, there was no consensus on the definition or management of marginally/borderline resectable tumors. The criteria in use at the M.D. University of Texas Anderson Cancer Center for BR-LA-PDAC in 2005 [14] were updated in 2006 [15] and are reported in Table 1.

Subsequently, three subgroups of BR-PDAC patients were defined based on the following clinical and radiographic characteristics: Type A: patients with borderline resectable tumor anatomy (see Table 1); Type B: patients classified with BR-PDAC due to the concern for possible extrapancreatic metastatic disease and those with known N1 disease from either pre-referral laparotomy or endoscopic ultrasound-guided fine-needle aspiration; and Type C: patients with BR-PDAC due to a marginal Eastern Cooperative Oncology Group performance status 3 (ECOG 3) or with a better performance status and significant pre-existing medical co-morbidity thought to require protracted evaluation, thereby precluding immediate surgery [15]. Given that with available surgical techniques, patients with BR-PDAC were at a high risk of positive margin resection, preoperative systemic chemotherapy and local-regional chemoradiation were used to maximize the potential of an R0 resection and prevent R2 resections [14,15,16]. The following definition of BR-PDAC was reached in 2009 by the American Hepato-Pancreato-Biliary Association (AHPBA), the Society of Surgical Oncology (SSO), and the Society for Surgery of the Alimentary Tract (SSAT) [17,18,19,20]: Tumors that are considered localized and resectable should demonstrate the following: a. no distant metastases; b. no radiographic evidence of superior mesenteric vein-portal vein (SMV-PV) abutment, distortion, tumor thrombus, or venous encasement; and c. clear fat planes around the celiac axis, hepatic artery (HA), and superior mesenteric artery (SMA). Tumors that are considered borderline resectable include the following: a. no distant metastases. b. venous involvement of the SMV-PV demonstrating a tumor abutment with or without an impingement and narrowing of the lumen, an encasement of the SMV-PV, but without an encasement of the nearby arteries, or a short segment venous occlusion, resulting from either a tumor thrombus or encasement but with a suitable vessel proximal and distal to the area of vessel involvement, allowing for a safe resection and reconstruction; c. a gastroduodenal artery encasement up to the HA with either a short segment encasement or a direct abutment of the HA, without extension to the celiac axis; and d. a tumor abutment of the SMA not >180° of the circumference of the vessel wall. The original AHPBA/SSO/SSAT classification has been modified by the International Study Group on Pancreatic Surgery (ISGPS) [21] and the National Comprehensive Cancer Network (NCCN) [22]. According to these definitions, BR-PDAC includes tumor findings associated with (1) distortion, narrowing, or occlusion of the SMV-PV, but with the technical feasibility of reconstruction; (2) a semi-circumferential abutment (≤180°) of the SMA; and (3) tumor contact with the HA without extension to the celiac axis. LA-PDAC is instead characterized by (1) a more extended involvement of the afore mentioned vessels, (2) any tumor involvement of the aorta or inferior vena cava, or (3) the involvement of the SMV-PV without the feasibility of venous reconstruction. Based on a symposium held during the 20th meeting of the International Association of Pancreatology (IAP) in Sendai, Japan, in 2016, a consensus was reached on issues related to BR-LA-PDAC (Table 2). Patients with BR-LA-PDAC were defined according to three distinct dimensions: anatomical (A) (Table 2), biological (B), and conditional (C) (Table 3) [23].

The rationale for IAP’s definitions was no longer only the high risk of a positive margin resection but also the tumor’s biology and the individual patient’s conditions playing a fundamental role in determining surgical outcome. However, it must be considered that about 10% of the population, due to the lack of the necessary Lewis glycosyltransferase, cannot express CA 19-9, which therefore cannot be used for prognostic purposes [24].

A consensus statement has been reached by a panel of experts on better imaging procedures, and their limits, to be performed before and after neoadjuvant therapy (NAT) in BR-PDAC patients and on the use of the NCCN definition of BR-PDAC [25]. Since then, several retrospective or prospective, completed or still ongoing studies have used study-specific criteria or even grouped BR-PDAC with either resectable or LA-PDAC, complicating the interpretation of results and demonstrating the need for standardization [26,27,28,29,30,31,32]. Notably, there are differences between different clinical guidelines [22,33], and the guidelines themselves have changed over time, with the NCCN undergoing frequent modifications and updating. The most used guidelines to define BR-PDAC and LA-PDAC are those of the NCCN [22]. A recent study by Badgery et al. [34] added to the differences between the different guidelines (MD Anderson, AHPBA/SSAT/SSO, NCCN, IAP and Intergroup pilot study classifications) [15,17,18,19,20,22,23,35] the possible variability in the interpretation of CT images by radiologists and surgeons. An international group of 77 physicians (33 hepato-pancreatobiliary surgeons and 44 radiologists) were asked specific questions, based on the International Consensus Guidelines [23], on the CT images of 30 patients believed to have BR-PDAC during multidisciplinary meetings. There was a high degree of variability in the assessment of resectability status among the reviewers, and in none of the 30 patients was the assessment of resectability unanimous [34]. The consistency of clinical decision making for patients with PDAC is therefore questioned by authors, suggesting a “central review for studies on neoadjuvant or adjuvant approaches in the future, as well as ongoing quality control initiatives, even amongst experts in the field” [34].

### 2.2. Anatomical Definition Problems

Until recently, the anatomical definition of resectability prevailed over the biological evaluation of PDAC without considering that it becomes a systemic disease early and, consequently, adequate local control of the disease has a limited effect on survival [36]. Indeed, in the four-arm, multicenter, randomized phase-2 trial (ESPAC5) [37], the R0 margin rate was higher in the group receiving neoadjuvant capecitabine-based chemoradiation (three out of eight, 37%) than in the neoadjuvant gemcitabine plus capecitabine and FOLFIRINOX groups (two out of eleven for both, 18%), while the opposite was true for one-year overall survival (OS) (60% and 78–84%, respectively). Chawla et al. [38] identified the site of first recurrence from a retrospective cohort of patients from 2011 to 2017 and from two prospective cohorts. Distant metastasis was the first site of recurrence in the vast majority (>80%) of those patients who initially, or after NAT, underwent an R0 or R1 resection for PDAC. Although NAT may be important in selecting those patients who may benefit from a curative-intent operation and increases the R0 resection rate from 57.6% of upfront surgery (UFS) to 79.2% of the retrospective cohort and 96.9% of the prospective cohort, it does not alter the pattern of recurrence [38]. In addition, the effect of an R1 resection on final pathology does not necessarily portend locoregional recurrence; these patients are also likely to manifest first recurrence at distant sites. The use of NAT may control the occult microscopic sites of distant disease, and in this setting, it will likely also control locoregional disease. Taken together, occult micrometastatic disease, rather than locoregional disease, is the driving force behind recurrence and survival in PDAC [38]. The authors’ “systemic disease from the beginning” hypothesis about PDAC is not new, as many surgeons view margin status as irrelevant in this disease since systemic failure determines the outcome in most patients [39,40,41].

A debate on the usefulness of a thorough examination of the resection margins (RM) in evaluating the prognosis of patients with PDAC undergoing surgical resection began thirty years ago [42]. Tumor involvement at the RM (R1) was reported in 37/72 patients (51%), with the peripancreatic soft tissue (27/37) being the most commonly involved margin. The R1 rate remained between 23.6 and 50.0% [43] until Verbeke et al. [44], applying a standardized protocol and R1 resection definition as a tumor within 1 mm of the RM, reported an R1 incidence of 84.6%, and the level remained >70% in patients undergoing UFS [45,46,47]. Stratifying the minimum clearance of the RM by 0.5 mm increments, Chang et al. [48] showed that it was not until the RM was clear by more than 1.5 mm that the optimal long-term survival was achieved. Jamieson et al. [47] reported a worse survival (*p* < 0.001) for the R1 group (n. 109) than for the R0 group (n. 38), but, after considering separately the R1_transection_ margins (pancreas, bile duct, stomach, or duodenum; n. 61) and the R1_mobilization_ margins (where two adjacent organ surfaces have been simply separated by developing embryological planes; n. 48), the significant difference in the survival of the R1_mobilization_ group compared with the R0 group disappeared (*p* = 0.52). The “systemic disease from the beginning” hypothesis [38] explains this better than the initial local progression followed by distant metastases, according to the results of Ghaneh, Chawla, and Jamieson [37,38,47]. In any case, achieving R0 resection with a margin of at least 1mm should be a primary goal in the surgical treatment of resectable and BR-LA-PDAC after NAT [49]. Oba A et al. [50] reported the results of a new nomogram based only on objective preoperatively available data and excluding any of the existing subjective terminology (i.e., borderline resectable and locally advanced). The obtained total scores were used to classify patients of the derivation cohort into three groups: patients with less than 190 total points, 190 to 394, and 395 or more, representing a group with a good, intermediate, and poor prognosis, respectively [50]. The assumption that the anatomical characteristics used to define resectable, BR, and LA PDAC are a marker of cancer behavior and that all patients who share a common anatomical profile should undergo the same sequence of treatments omits to consider the biological features of PDAC [50,51,52]. Furthermore, thanks to a study on the gene expression of PDAC, groups of patients with a low, intermediate, or high risk of invasion and metastasis, propensity for metastatic recurrence, and poor OS were identified [53,54,55,56,57,58]. Therefore, the rationale for choosing between UFS followed by adjuvant therapy and NAT followed by resection and adjuvant therapy for patients with BR-PDAC needs to be clearly defined.

### 2.3. UFS or NAT/NACRT (Neoadjuvant Chemoradiotherapy)?

According to current NCCN guidelines, NAT is considered the standard of care for patients with BR-PDAC, high-risk patients with resectable disease, and selected patients with LA-PDAC [59]. Acceptable regimens include FOLFIRINOX, gemcitabine/albumin-bound paclitaxel, and gemcitabine/cisplatin [59]. Primary treatment with FOLFIRINOX compared with gemcitabine-based chemotherapy appears to provide a survival benefit for patients who are ultimately unresectable. For patients that undergo surgical resection, the outcomes are similar between GEM+ and FOLFIRINOX when delivered in the neoadjuvant setting [60]. Better survival after NAT than after UFS in BR-PDAC patients has been reported by several authors [37,61,62,63,64,65,66]. Among these, Jung et al. [64] included 19 publications with 21 datasets in their analysis, involving a total of 2906 patients (NAT, 1516; UFS, 1390). The mean resection rate was higher after UFS (81.4%) than after NAT (67.9%), but the R0 resection rate was higher (81.7% vs. 58.7%), and the lymph node (LN) positivity rate was lower (46.4% vs. 78.0%) after NAT than after UFS. There are several ongoing prospective studies on NAT for resectable and/or BR-PDAC before radical resection [67].

However, there are still several open issues regarding the use of NAT in BR-LA-PDAC patients. First, there is the issue of whether an individual patient’s significant response to NAT can be predicted. The reported lower resectability rate after NAT compared to UFS [64] is, at least in part, due to disease progression, which became unresectable after the completion of NAT. According to Dong et al. [68], in addition to enabling the treatment of early micro-metastatic disease and improving OS, NAT is useful for selecting patients who will progress during treatment before proceeding to surgery, thus saving a futile operation. It is hoped that the goal of avoiding futile interventions due to ineffective NATs will be progressively reduced by an improvement in the selection of effective NATs and in the management of the UFS/NAT sequence from future evidence-based studies. Nahm et al. [69] explored the ability of a triple biomarker panel (S100A4, Ca-125, and mesothelin) to predict genetic PDAC subtypes, clinical phenotypes, and the optimal treatment strategy (NAT vs. UFS) in resectable and borderline resectable PDAC. A triple-negative biomarker status (how-risk PDAC phenotype) was associated with the non-squamous subtypes of PDAC and with worse survival outcomes if resection is delayed due to NAT treatment. In contrast, a triple-positive biomarker status (high-risk PDAC phenotype) was associated with the squamous subtype of PDAC and better survival outcomes with NAT treatment prior to resection [69]. According to Nitschke et al. [70], mutant-KRAS detection in the peripheral blood, and even better, in the portal vein, is an independent adverse prognostic marker in curative and palliative PDAC patients and could be an effective novel tool for identifying prognostic borderline patients, guiding future decision making on NAT despite anatomical resectability. Oshima et al. [71] evaluated the association between the three major genetic mutations (*P16*, *TP53*, and *SMAD4/*DPC4) associated with PDAC and their malignant behavior in 43 patients with resectable PDAC and 41 patients with BR-PDAC that underwent EUS-FNA before NAT. The three main genetic mutations were evaluated by immunohistochemistry both preoperatively in the EUS-FNA sample of 84 patients and in the resected PDAC sample of 71 patients, with 13 patients unresectable at the end of NAT. The pre-treatment abnormal labeling of p53 in a EUS-FNA specimen was associated with a lower resection rate and an early recurrence in resectable or BR-PDAC cases. Unfortunately, the number of patients included in the study was not sufficient to draw definitive conclusions; moreover, no patient underwent UFS, and therefore, it is not possible to state that NAT first was the best possible treatment for all patients.

Pathological studies of post-NAT pancreatic resection specimens have shown that only 12.6–18.6% of PDAC patients demonstrate a pathological complete or near-complete response to NAT, which is associated with improved survival, while the majority part of the patients (>80%) demonstrate a moderate or minimal response to NAT and a poor survival [72]. Furthermore, it is not uncommon to observe a heterogeneous intratumor response to NAT in different areas of the same treated tumor, with some areas showing a complete or near-complete response and others showing minimal or no response [72]. Currently, the choice of the therapeutic sequence (NAT first/UFS first) for the individual patient is mainly based on the statistical criterion of the greater efficacy of NAT compared to UFS [37,61,62,63,64,65,66]. Making this choice based on the evidence provided by the predictive biomarkers of the prognosis of the individual patient with PDAC represents a challenge. The initial evaluation via immunohistochemistry of the EUS-FNA sample and/or liquid biopsy (free-circulating DNA, circulating KRAS ctDNA, exosomes, microRNA, circulating tumor cells (CTC), circulating cancer-associated macrophage cells, biomarkers, and peripheral blood lymphocytes [70,73,74,75,76,77,78,79]) can help identify which biomarkers may be useful for trying to overcome the challenge. The main limitation of liquid biopsy is its lack of sensitivity and accuracy in identifying various types of PDAC compared to tissue biopsy. In addition, the ability to detect biomarkers in a liquid biopsy is difficult because CTCs, ctDNA, and RNA are relatively scarce compared to other blood components. Furthermore, there are still no standard methodologies for separation, enrichment, and detection [73]. The most significant advantage of liquid biopsy over tissue biopsy is its ability to monitor disease progression and treatment efficacy longitudinally in “real time”. However, liquid biopsy is not yet considered a standard means of confirming or diagnosing various diseases, including cancer [73,80]. Therefore, we are still in the phase of defining the role of different biomarkers in the evaluation of the prognosis of PDAC and its possible response to NAT, while there is still no generally accepted panel of immunohistochemical and/or biological biomarkers predictive of disease evolution. The current approaches to precision medicine in PDAC are often limited by the inadequate availability of material to evaluate the predictive biomarkers of responses to NAT [81]. The ability to rapidly establish and expand PDAC-derived three-dimensional tissue cultures has been proposed as a feasible strategy to support precision medicine. These patient-derived organoids (DOPs) reliably demonstrate the molecular characteristics of disease in vivo when genotyped in the research setting [81]. Recently, a biomarker based on a novel multigene FFX-ΔGEP score using targeted immune-gene expression profiling was developed to predict the lack of FOLFIRINOX response in PDAC patients after only one cycle [82]. Pending biomarkers that can predict responses to different types of NAT, which would enable choosing the most suitable NAT for the individual patient, the ability of the FFX-ΔGEP multigene score to recognize early NAT ineffectiveness, if confirmed by further studies, would be an important step in the direction of precision medicine. Patients with LA-PDAC also typically undergo chemotherapy for palliative purposes. For these patients too, the choice of the most suitable chemotherapy and/or the early verification of its possible ineffectiveness are important. Furthermore, patients with LA-PDAC can be down staged to BR-PDAC or resectable PDAC in some cases.

### 2.4. Resectability after NAT/NACRT

Preventing an unnecessary and risky R1 pancreatic resection is the main goal of restaging after NAT. MDCT is commonly used to assess the initial resectability of PDAC, with an accuracy of 73–97% for predicting R0 resection in patients undergoing UFS [83]. Conversely, an MDCT assessment of R0 resectability after NAT is thought to underestimate pathological responses [84,85,86]. A recent meta-analysis showed that the ordinary criteria used to interpret the MDCT results after NAT to select candidates for R0 resection are not sensitive (45% of summary statistics) but highly specific (85% of summary statistics), although with substantial study heterogeneity [87]. Alternatives to MDCT include magnetic resonance imaging (MRI). Similarly to MDCT, MRI utilizes time-contrast images to visualize the arterial and portal venous anatomy. Yang et al. [88] showed that MRI and CT have similar performance in assessing PDAC tumor size before and after NAT. However, dynamic MRI is not commonly performed and should only be attempted by experienced centers due to difficulties in reproducibility and standardization [89]. Provided that resectable or borderline resectable tumors on post-NAT CT were considered eligible for resection, the sensitivity and specificity of the CT-determined resectability were 95.2% (95% CI, 89.1–98.4%) and 8.7% (95% CI, 1.1–28.0%), respectively [90]. There are several reasons why conventional imaging is unreliable in accurately predicting an R0 resection after NAT. The first concerns the inflammatory and fibrotic reaction of the perivascular tissue or its sterile necrosis (following a complete pathological response) induced by NAT, which can be indistinguishable from neoplastic infiltration. The second concerns the possibility that an imaging-clean adipose plane can be restored by NAT, providing an indication of a possible R0 resection that turns out to be incorrect due to the presence of a residual microscopic infiltration of the perivascular region. In the first hypothesis, the forecast of an R0 resectability would be erroneously reduced, and in the second, it would be erroneously increased [89]. The NCCN guidelines have introduced customized criteria for PDAC resection after NAT, where radiographic findings, unless demonstrating clear disease progression, are no longer the only criteria used to propose exploratory surgery in the presence of other patient data showing clinical improvement and at least stable or decreased CA 19-9 [59]. According to Jang et al. [91], the NCCN criteria significantly improved the sensitivity for R0 prediction (from 26.9% to 87.5%; *p* < 0.001), that is, it overcame the underestimation of resectability that occurs on post-NAT CT. On the contrary, the NCCN criteria showed higher specificity for R0 prediction than CT-determined resectability when both resectable and borderline resectable tumors were considered eligible for resection (21.7% vs. 8.7%; *p* = 0.375), which could be helpful for reducing undesirable surgical explorations (extensive surgery with a non-R0 resection) after NAT [91]. A histopathologic assessment of post-NAT pancreatectomy specimens to determine the diagnosis, tumor grade, margin status, tumor size, lymph node status, presence of lymphovascular, or perineural invasion, as well as the tumor’s response to NAT is important in predicting the prognoses of PDAC patients. In particular, the results of the pathological assessment of tumor response to NAT were reported based on six different systems: the College of American Pathologists (CAP) system [92,93,94], the Evans grading system [92,93], the HTRG scheme [95], the MD Anderson Cancer Center scheme [93], the Royal North Shore (RNS) system [93], and the integrated pathologic score (IPS) [96]. Better results with no viable tumor or minimal residual carcinoma in 10.9–27.9% of patients, intermediate results in 44.5–73.1%, and worst results in 8.1–30.5% of patients were reported (Table 4) [92,93,94,95,96].

Despite the striking differences in tumor response grading, an important proportion of patients achieve little or no response to NAT, with survival after surgical resection not significantly different from that of similar unoperated patients. Recognizing at the end of the NAT the patients in whom it was of little, or no effect could enable a more objective evaluation of the usefulness of a possible surgical resection with respect to further neoadjuvant treatment or a better supporting care. Currently, CA19-9 is undisputedly the most important surrogate marker of PDAC biology [59,97,98,99,100,101,102,103,104]. Unfortunately, approximately 10% of the population cannot express CA 19-9 [24,97], and this is “C”onsistently normal” in around 18% (30/166) of patients undergoing NAT [98]. Several CA 19-9 post-NAT preoperative cut-off values associated with improved OS have been proposed: normalization [86,101,102], decrease > 50% or normalization [103,104,105], decrease > 85% [99,106], >40% [107], >30% [108], and value < 500 U/mL [109]. According to Seelen et al. [110], 70.2% of resected LA-PDACs after NAT recurred after a median follow-up of 28 months. The optimal cut-off for recurrence-free survival to differentiate between early (n. 52) and late (n. 66) recurrence was six months (*p* < 0.001). OS was significantly shorter (8.4 vs. 31.1 months, *p* < 0.001) for the early recurrence group. A preoperative predictor for early recurrence was postinduction therapy, with CA 19-9 ≥ 100 U/mL (*p* = 0.001) [110]. Furthermore, a CA19-9 decrease of ≥60% following induction chemotherapy as the optimal response cut-off in patients with LA-PDAC is an independent predictor for OS when CA19-9 is increased at baseline, and ≥40% is the minimum cut-off demonstrating survival benefit [111]. Despite the variability in the cut-off values reported by several authors and the need for their possible standardization, CA 19-9 is a very useful tool to evaluate the response to NAT in PDAC and could also be used to guide the overall duration of the neoadjuvant treatment. However, it cannot be the only tool used for an evaluation as a significant proportion of patients with PDAC may not secrete or have normal CA 19-9 values [24,97,98]. As previously reported, it is hoped that liquid biopsy will define biological biomarkers predictive of disease evolution to be used, together with CA 19-9, for assessing treatment response and prognosis in patients with PDAC [73,74,75,76,77,78,79]. ^18^F-Fludeoxyglucose positron emission tomography (^18^F-FDG-PET) has been increasingly used in the differential diagnosis and prognostic evaluation of PDAC since the early 1990s [112]. However, despite the considerable evolution of equipment with the appearance of PET/CT [113], its progressive updating [114], and, recently, PET-MRI [115], the role and usefulness of ^18^F-FDG-PET/CT/MRI in the management of patients with PDAC are still considered “under development” by current guidelines [115]. The NCCN consensus guidelines state that ^18^F-FDG-PET/CT may be considered after the formal pancreatic CT protocol in high-risk patients to detect extra-pancreatic metastases [59].

According to the recently revised Clinical Practice Guidelines for Pancreatic Cancer of the Japan Pancreas Society, “Positron emission tomography is not recommended as a diagnostic or qualitative diagnostic tool in patients with suspected pancreatic cancer”, but it “is recommended in patients with suspected distant metastasis, as PET is more specific than CT for the diagnosis of distant metastasis” [116]. Furthermore, ^18^F-FDG-PET/CT is not considered by the European Society for Medical Oncology (ESMO), the ISGPS, and the Italian Association of Medical Oncology (AIOM) guidelines [21,117,118]. According to Heinrich et al. [119], ^18^F-FDG-PET/CT represents an important staging procedure prior to pancreatic resection for PDAC since it significantly improves patient selection and is cost-effective. Furthermore, the PET-PANC study showed that, in patients with primary suspected pancreatic malignancies, non-therapeutic laparotomy was avoided in 21% of patients [120]. Furthermore, several authors have recently evaluated positively the role of ^18^F-FDG-PET/CT in the management of PDAC [121,122,123,124,125]. Based on Ghaneh’s study, ^18^F-FDG-PET/CT was added to the staging recommendations in the UK NIHCE guidelines [126]. According to Itchins et al. [127], little data exist on the usefulness of ^18^F-FDG-PET/CT in PDAC treated with NAT. However, there is growing evidence that the standardized uptake value (SUV) is related to the aggressiveness of tumors, and it has been hypothesized that the SUV may predict R0 resection [128]. It has been reported that ^18^F-FDG-PET/CT would be very useful for monitoring the NAT effect on FDG-avid tumors, which are defined as those with a maximum SUV (SUV_max_) > 5 [101]. A complete metabolic response (CMR) after NAT was defined as an FDG uptake indistinguishable from the background in patients with FDG-avid tumors pre-NAT at baseline [101,102,129]. Importantly, ^18^F-FDG-PET/CT could also predict the histological response of the surgical specimen, which is the strongest evidence of the NAT effect [102]. Barreto et al. [130] reported that patients with a CMR had a 100% reduction in SUVmax, while those who had a pathological partial response had a 61% reduction in SUVmax. A minor metabolic response was defined as persistent or higher FDG activity than the adjacent background tissues and compared with baseline ^18^F-FDG-PET/CT, if available [102]. According to the RECIST 1.1 criteria [131], a partial response corresponds to a ≥30% decrease in the SUV [129,132]. An SUV_max_ < 4.5 achieved during NAT was considered sufficient to proceed with surgical resection, while the persistence of values ≥ 4.5 and CA 19-9 > 37 U/mL suggested a continuation with NAT [133]. According to Tabata et al. [134], a high initial SUV_max_ would predict the effectiveness of NAT, while according to Benz et al. [132], the level of the initial SUV_max_ would have no influence on the response probability. It is important to underline that the responders were only 5/28 and 6/23 patients, respectively, in line with the level of pathological complete or near-complete response to NAT. Benz et al. [132] suggested performing three ^18^F-FDG-PET /CT scans: before NAT, after 4 weeks of NAT, and at the end of NAT, allowing them to detect early non-responders and to consider patients with a >30% reduction in SUV_max_ after NAT as partial responders. Furthermore, digital PET/CT imaging allowed for the detection of small-volume liver metastases in 14% of patients, whereas in retrospect, only 3% could have been detected through MDCT [114]. Unfortunately, ^18^F-FDG-based PET/CT has some limitations due to the high physiological uptake in many normal tissues (brain, salivary glands, vocal cords, myocardium, and urinary tract), which hinders the detection of tumor lesions; a low uptake in some tumor types; insufficient lesion-to-background signal ratios in smaller lesions; a limited role in detecting LN involvement; and a lack of specificity, such as in inflammatory pancreatic disease and increased uptake seen in tumor-associated pancreatitis, which could potentially lead to unnecessary pancreatic resections [135,136,137,138,139,140]. The staging problems of PDAC, even in the current era of high-quality imaging, are also evidenced by the results of a staging laparoscopy, performed in 1004 patients between 2017 and 2021, that resulted in a change in management in one in five patients [141]. The need for better preoperative diagnostics, especially after NAT, due to the previously exposed imaging problems, has led to the research and development of tumor-specific tracers that can provide alternative solutions for a more accurate staging and better monitoring of therapeutic response. The Fibroblast Activating Protein Inhibitor (FAPI) is a tumor-specific tracer on which we already have important clinical studies. This choice is because, in PDAC, more than 90% of the tumor volume consists of cancer-associated fibroblasts (CAFs) that are associated with the promotion of tumor growth, tissue invasion, metastasis development, immune escape, and resistance to therapy [142,143,144]. Considering the high expression of the FAP on the cell surfaces of CAFs and its limited expression in normal tissue, PET/CT imaging of CAFs with radiolabeled FAP inhibitors is an active field in nuclear medicine [137]. ^68^Ga- and ^18^F-radiolabeled FAPI variants (including FAPI-04, FAPI-46, and FAPI-74) have produced promising results in the diagnosis of various cancers, [135,136,137] and, in particular, in the diagnosis of PDAC [138,139,140]. The measurement of the intracellular levels of phosphorylated thymidine has been postulated to be an accurate method for estimating cellular growth. Fluorinated thymidine analogues, such as ^18^F-Fluorothymidine (^18^F-FLT), are trapped as radiolabeled phosphates into the cell, allowing for the uptake of the radiolabeled thymidine analogues as a surrogate measure of cellular proliferation. ^18^F-FLT-PET/CT imaging is potentially superior to ^18^F-FDG-PET/CT, as ^18^F-FLT uptake is not affected by inflammation or hyperglycemia [139,145]. Several other tumor-specific tracers are under development and evaluation. Integrins are proteins that facilitate the adhesion of cells to the extracellular matrix of polypeptides. Integrin αυβ6 promotes the invasive phenotype of PDAC by modulating the proliferation, survival, migration, and invasion of both the cancer cells and their microenvironment [139,146]. It appears to be an integrin important for the detection of PDAC and for distinguishing it from pancreatitis [147]. No relevant uptakes of ^68^Ga-Trivehexin (^68^Ga-labeled trimerized αυβ6-integrin selective nonapeptide) are seen in other organs and tissues, except excretion-related in the kidneys and urinary tract, which do not compromise tumor visualization in the investigated settings [147]. Tip-like endothelial cells (ECs) are the main differential subcluster of ECs between tumors and normal tissues. Tip-like ECs represent an activated EC subcluster that can promote tumor angiogenesis and influence the tumor immune microenvironment. The presence of a high proportion of tip-like ECs correlates with poor clinical outcomes in multiple cancer types [148]. A prostate-specific membrane antigen (PSMA) can be used as a specific marker for tip-like ECs, which confirms the rationale for its use as a target for the diagnosis and treatment of non-prostate cancers as well [148]. Krishnaraju et al. [149] showed improved diagnostic accuracy with ^Ga^68-PSMA-PET/CT compared to ^18^F-FDG-PET/CT in a study among 40 patients with pancreatic lesions (21 benign and 19 malignant).

In conclusion, the evaluation of resectability after NAT is mainly based on NCCN guidelines [59]. Despite the increased efficacy of NAT after the introduction of FOLFIRINOX and Gemcitabine-based chemotherapy, this better pathological response to NAT is still limited to 10.9–27.9% of patients, as reported in Table 4. According to De Simoni et al. [150], total neoadjuvant therapy, intended as induction chemotherapy followed by radio-chemotherapy, demonstrated a potential superiority to NAT without radio-chemotherapy in terms of the oncological and pathological outcomes, even if the main differences seem to depend on the induction chemotherapy regimen. Several problems still exist: 1. how to predict early recurrence after radical resection to identify patients with poor prognoses and avoid unnecessary surgery [110,151,152]; 2. how to select the most active chemotherapy regimen (using DOPs [81] or other procedures) and predict a lack of response to FOLFIRINOX, when used, after only one cycle [82]; and 3. which tumor-specific tracer can be used for PET/CT to provide more accurate staging and better monitoring of therapeutic response.

A multicenter prospective observational recruiting, study (NCT05356039) will evaluate survival, quality of life, exploration, and resection rates in BR-LA-PDAC patients undergoing oncological treatment. The estimated study completion date is May 2028.

## 3. Synchronous Oligometastatic PDAC

It is estimated that approximately 50% of PDAC patients have metastatic disease at presentation [153,154]. Metastatic PDAC is generally considered unresectable according to international guidelines [21,22,117,118,126], regardless of the size and number of distant metastases. Palliative systemic chemotherapy has been the standard of care for these patients, and long-term survival remains limited, with a median OS of 8.5–11.1 months and progression-free survival of 3.3–6.4 months [155]. However, in recent years, the careful follow-up of PDAC patients has shown that, among metastatic patients, there may be a subgroup with an intermediate disease between localized disease and systemically widespread disease. The definition of this subgroup of patients was first introduced by Hellman and Weichselbaum in 1995 [156] under the term “oligometastatic” cancer, in which metastases are small in number and confined to a single or limited number of organs “because the facility for metastatic growth has not been fully developed and the site for such growth is restricted”. In such cases, curative treatment may still be possible. The simultaneous surgical resection of the primary tumor and liver metastases has shown a significant improvement in long-term survival for patients with colorectal, neuroendocrine, and gastric cancers who had previously been excluded from surgical treatment [157]. The existence of an oligometastatic phase of pancreatic cancer is not yet widely accepted, and a unique and widely accepted definition of an anatomically “limited disease” is still lacking [158]. The number of liver metastases set by several recent authors required to consider a PDAC “oligometastatic” varied between ≤3 [159,160,161], ≤4 [153,154,162,163], ≤5 [164,165], and 1–21 [166]. In contrast to the above reported malignancies (colorectal, neuroendocrine, and gastric cancers), oligometastatic PDAC is generally considered a contraindication to surgical resection, even if a few visible metastatic lesions are limited to one organ and are easily resected. Twelve literature reviews on the subject carried out since the 2017 [155,157,158,167,168,169,170,171,172,173,174,175] reported data from 38 articles overall. After considering the results of each of the 38 articles reported by the twelve reviews, excluding articles reporting duplicate (6) or non-topic (1) results, and considering the presence of two reviews, one article based on data from the Surveillance, Epidemiology and End Results program (1977–2001) and another from the National Cancer database (2010–2015), we estimated that approximately 1400 patients with liver-only oligometastases undergoing a simultaneous resection of the primary tumor and metastatic disease were reported overall. Due to the variety of criteria used to choose a surgical treatment, the variety of chemotherapy treatments used by different authors, and the variety of intervals between diagnosis and surgical resection, the twelve reviews reached different conclusions. Two were in favor of surgical resection due to its additional survival benefit in the medium term [167,171], one was largely inconclusive due to its limited study population and the heterogeneity of inclusion criteria among studies [173]; two interpreted the positive results of surgery as surrogate markers of favorable tumor biology, as appropriate tumor selection remains a challenge [155,172]; four were in favor of surgery only after an adequate response to NAT [157,158,168,170], even more so if this was associated with adjuvant chemotherapy [168], but also expressed their concern about patient selection and the timing of surgery [157]; and finally, three were in favor of surgery but underlined the difficulty in defining the appropriate time for surgery and chemotherapy [169] and the difficulty in selecting patients due to the inadequate selection capacity of the available biomarkers [174,175]. Seven further articles on the topic have recently been published. Three reported improved survival after surgical resection for highly selected oligometastatic PDAC patients [162,171,176], but one of them required that surgical treatment must not impair a patient’s reception of adjuvant chemotherapy [162]. Four reported improved survival after NAT and surgical resection [153,161,177,178]. Both the results of the twelve reviews [155,157,158,167,168,169,170,171,172,173,174,175] and of the seven recently published articles [153,161,162,171,175,176,177,178] are influenced by a selection bias due to the variety of criteria with which surgical resection patients were selected, as demonstrated by Table 5 [153,159,160,166,171,178,179,180,181,182,183,184,185,186,187,188,189] and Macfie’s Table 3 [152].

From the two tables, it appears that the prevailing criterion was the anatomical one, aimed at obtaining an R0 resection for both the primary tumor and the metastases (82.6% of the articles), and was even the only criterion used by 65.2% of the articles and, in particular, by 78.6% of those published up to and including 2020. The biological criterion was considered by only 34.8% of the articles and was based almost exclusively on a CA 19-9 evaluation. Finally, the oncological criterion was based on a satisfactory morphological and/or biological response to NAT and was considered by only 26.1% of the articles. Considering 38 articles on the topic (31 included in the 12 reviews [155,157,158,167,168,169,170,171,172,173,174,175], after excluding duplicate or non-topic publications, and 7 recent articles not included in the reviews [153,161,162,171,175,176,177,178]), adequate data on NAT (useful/not useful), the interval between diagnosis and surgical resection after NAT (so called immortal time [154]), CA 19-9, and the oncological criteria used to propose synchronous surgical resection were reported by 21, 7, 23, and 14 articles. Of the 21 articles reporting data on NAT, almost all published after 2015, 76.2% reported satisfactory results, while it was considered useless by 14.3% and its effect was not specified by 9.5%. The modest interest in using NAT in patients with synchronous oligometastatic PDAC contrasts with the great attention paid to the choice of NAT for its potential benefits in patients with BR-LA-PDAC [37,61,62,63,64,65,66] and with the results of studies demonstrating increased OS in patients with metastatic PDAC who underwent treatment with either FOLFIRINOX or Gemcitabine-based chemotherapy [190,191]. Only 7 of the 21 articles reporting data on NAT provided data on the interval between the diagnosis and surgery [153,160,161,189,192,193,194], but none of them considered this interval for the unresected group selection to prevent immortal time bias in the statistical analysis [154]. CA 19-9 was considered useful only by 16 (69.6%) of the 23 articles that reported it, and by 14, it was used to evaluate the oncological response to NAT on the basis of different criteria: normalization: 2; decrease > 50%: 4; unspecified reduction: 2; exclusion if persistently high: 1; and maximum value within which to consider surgical resection: 5. The latter value was defined a priori by three (<400, <400, <1000 IU/mL) and a posteriori by two (<400 and <500 IU/mL). CEA and CA 125 were both reported as useful by one article. Apart from the fact that the CA 19-9 measurement may be useless in an important percentage of cases [24,97,98] and that its post-NAT decrease or normalization cannot be associated independently with survival [161], the CA 19-9 value may only be useful for estimating tumor burden and not tumor metabolic capacity or ability to metastasize. Only 3 out of 38 articles reported data on ^18^F-FDG-PET/CT, but it was only used to exclude further metastatic disease, while it was increasingly used for prognostic evaluation in patients with BR-LA-PDAC [101,102,129,130,131,132,133,134]. Takeda et al. [160] identified patients with the best prognosis after surgical resection (19.9 vs. 8.3 months, *p* < 0.001) using a prognostic index based on four parameters (age < 70 years, performance status of 0, modified Glasgow prognostic score of 0, and carbohydrate antigen 19-9 < 1000U/mL). Li et al. [177] developed a nomogram based on a series of metastatic, therapeutic, and pathological features to obtain a preliminary prediction of the impact of surgical treatment on the long-term prognosis of patients with metastatic PDAC. However, both studies were based on retrospective data without prospective validation. Furthermore, none of the other authors of the 38 articles made any effort to identify additional prognostic parameters, such as PDAC subtype, mutant KRAS detection in blood, and liquid biopsy [69,70,71,72,73,74,75,76,77,78,79], to improve the comparison of the long-term results of patients undergoing UFS, a surgical resection after NAT, or chemotherapy only. Peritoneal metastasis is generally considered a systemic metastasis that contraindicates surgical resection, while peritoneal oligometastatic disease has rarely been discussed and established. Sho et al. [175] reviewed seven articles reporting the results of 67 patients undergoing a resection of oligometastatic peritoneal PDAC. Studies up to 2010 reported an OS from the initiation of treatment ranging from 5.3 to 12.9 months, while it has ranged between 19.4 and 32.5 months in more recent studies. [175]. The criteria for surgery were good performance status, significant tumor shrinkage after NAT, decrease in tumor marker levels (CA 19-9), negative cytology results, and absence of peritoneal deposits on staging laparoscopy [175].

There are six ongoing clinical trials on the treatment of oligometastatic PDAC:

1. The Chinese Study Group for the Pancreatic Cancer (CSPAC)-1 trial (NCT03398291) is a recruiting multicenter, prospective, randomized phase-III control trial comparing synchronous resection after conversion chemotherapy to standard chemotherapy. The study’s completion is estimated on 1 June 2025.
2. The Hepatic Oligometastatic Pancreatic Cancer (HOLIPANC) study (NCT04617457) is a recruiting nonrandomized, multicenter, single-arm phase-II clinical trial for PDAC patients with hepatic oligometastases receiving neoadjuvant combination chemotherapy, where those with stable or responsive disease and a resectable primary tumor will undergo a synchronous resection of the tumor and hepatic metastases. The study’s completion is estimated on 30 September 2025.
3. The Hepatic Resection for Metastatic Pancreatic Cancer study (NCT02892305) is a recruiting, small interventional pilot study that will be conducted as a single-site study at Duke University Health System. A liver resection or ablation of oligometastases will be performed simultaneously with pancreaticoduodenectomy. The study’s completion is estimated on 30 June 2027.
4. The SCANPAN-1 trial (NCT05271110) is a Scandinavian, not-yet-recruiting, multicenter trial that will prospectively investigate a surgical resection of pancreatic cancer with synchronous hepatic oligometastases [195].
5. The Standard of Care Chemotherapy With or Without Stereotactic Body Radiation Therapy for the Treatment of Oligometastatic Pancreatic Cancer trial (NCT04975516) is a not-yet-recruiting phase-II trial (first posted: 23 July 2021) that studies the effect of standard of care chemotherapy with or without stereotactic body radiation therapy in treating patients with PDAC that has spread to a limited number of places in the body (oligometastatic).
6. A randomized phase-III trial of intravenous and intraperitoneal paclitaxel with S-1 versus gemcitabine plus nab-paclitaxel for PDAC with peritoneal metastasis (Japan Registry of Clinical Trials jRCTs051180199) is being performed in Japan. The study is aimed at confirming the efficacy of intravenous and intraperitoneal paclitaxel with S-1 compared to conventional systemic chemotherapy with gemcitabine plus nab-paclitaxel for peritoneal metastatic PDAC.

In summary, high-level evidence showing the benefit of the synchronous resection of PDAC and oligometastatic disease is still lacking. Until the results of these randomized controlled trials (RCTs) are available, the synchronous resection of PDAC and oligometastatic disease should only be performed within high-volume centers and in a research setting. After several RCTs demonstrating increased OS in patients with metastatic PDAC who underwent treatment with either gemcitabine-based chemotherapy or FOLFIRINOX [190,191] and better survival after surgical resection after NAT than after UFS [153,154,162,175,189], conversion surgery after satisfying response to NAT is considered the standard treatment of oligometastatic PDAC. However, an early choice of the most effective NAT thanks to DOPs [81] and predicting a lack of response to FOLFIRINOX, if used, after only one cycle [82] are of paramount importance considering the short median survival of M1 PDAC under a single-agent gemcitabine of 5.0 to 7.2 months [190]. The recent literature has shown that pancreatectomy with synchronous hepatic metastasectomy can be performed safely without a significant increase in perioperative morbidity and mortality [153,160,174]. However, molecular subtyping and information on PDAC biology, including an assessment of tumor metabolic capacity or ability to metastasize, can aid in treatment planning and patient selection. Many questions remain unanswered beyond the effectiveness of surgical resection in patients with stage IV oligometastatic disease: Which patients are most likely to benefit from surgery? What predictive factors can identify such patients? What is the most accurate method of estimating cell growth and the ability to metastasize of PDAC? How long should a patient be treated with NAT before resection is considered? Is there a role for stereotactic body radiotherapy and/or local ablative therapy to be associated with chemotherapy in the NAT of stage IV oligometastatic PDAC? Which metastatic sites (and how many) should be approached surgically? What pathological findings from resected specimens, other than an assessment of resection margins, will be useful in predicting long-term survival? Which biological markers can help to evaluate a patient’s long-term prognosis after an R0 resection of all visible disease?

## 4. Surgical Treatment of Metachronous Oligometastatic PDAC after Potentially Curative Resection

PDAC is an aggressive cancer with an overall 5-year survival rate that has increased to 12% thanks to more aggressive adjuvant and neoadjuvant treatment and surgical techniques [4]. Unfortunately, almost all patients will develop disease recurrence [196,197,198] even after R0 surgery [59,199]. Recurrence was diagnosed in 479 out of the 730 patients (65.6%) included in the ESPAC-4 trial; recurrence within 2 years of randomization occurred in 416 of 479 patients (86.8%) with recurrences, in 202 of 238 patients (84.9%) with a local recurrence, and in 214 of 241 patients (88.8%) with a distant recurrence. The overall median time to recurrence was 12.65 months [196]. The reported overall median time of recurrence varied between 8.0 and 12.65 months [197,200] but differed according to different recurrence patterns, being shorter for a liver-only recurrence (6.9 months) and increasing progressively with local and distant relapse (10.0 months), multiple relapse (11.7 months), local-only relapse (14.6 months), other (15.7 months), and lung-only relapse (18.6 months) [7]. Most patients after tumor relapse will undergo chemotherapy and or radiotherapy. The first report of a successful surgical treatment of locoregional recurrence without distant metastases was published in 1992 [201], but only in the 2000s did the first series from larger cohorts start to be reported [202,203]. True local recurrence is usually defined as the bed of the pancreatic margin, the pancreatic remnant, or the mesenteric root [201]. Several systematic reviews with meta-analyses and pooled analyses [204,205,206,207,208,209,210,211] have been published so far, which include a minimum of 50 [205] and a maximum of 1848 [206] patients undergoing a surgical resection of metachronous local or distant recurrences of PDAC (Table 6).

Unfortunately, none of the reviews reported separate data on the resection of early (<6 months) or late (>6 months) metachronous recurrences. Wright et al. [193] reported early disease progression (<6 months) after a surgical resection of tumor recurrence in 7/23 patients (30.4%). For these patients, the expected clinical benefit with surgical resection did not occur. Finding one or more markers that enable predicting which patients will experience an early progression after a resection of the recurrence would improve the surgical indication in these patients [193]. The better results after re-resection were achieved for isolated pulmonary recurrences, followed by isolated local (mainly pancreatic) recurrences, while the results after re-resection of liver and peritoneal recurrences were less satisfactory [206,207,208,209,210]. Only Serafini et al. [204] performed a statistical comparison between the survival curves of resected and unresected patients, showing a significantly better survival after re-resection than after chemotherapy/chemoradiotherapy. Unfortunately, the unresected patients included in four [212,213,214,215] (212 out of 255 patients) out of the six studies [212,213,214,215,216,217] included in the systematic review and meta-analysis were unresectable. However, the unresected patients in the other two studies [216,217] were resectable, although with a more complex or risky procedure [216]. Both studies showed significantly better survival (*p* < 0.012 and *p* = 0.014, respectively) for re-resected patients [216,217]. Of particular interest is that the reported mortality rate in the case of re-resection is between 1 and 2%.

An early diagnosis of metachronous PDAC metastases can bring the best therapeutic result and the highest chance for prolonged OS or even salvage therapy with curative intent [210].

However, none of the international guidelines [59,117,118,126] recommended a regular monitoring scheme. Recently, patient-derived xenografts (PDXs), created from surgical specimens, were reported as potentially valuable tools for planning the surveillance of pancreaticobiliary tumors, allowing for a timelier and more individualized intervention, which could improve patient outcomes [218]. CT and tumor markers (CA19-9) are now commonly used by many clinicians to monitor the postoperative follow-up of PDAC. However, according to Sperti et al. [219] and Schwarz et al. [220], tumor relapse after PDAC resection is detected earlier by ^18^F-FDG-PET/CT than by CT. Two systematic reviews and meta-analyses and one narrative review supported the usefulness of ^18^F-FDG-PET/CT in the follow-up of resected PDAC [221,222,223]. For CT, the pooled estimates for sensitivity were 0.70 (95% CI 0.61–0.78) and for specificity, 0.80 (95% CI 0.69–0.88). For ^18^F-FDG-PET/CT, pooled estimates for sensitivity and specificity were 0.88 (95% CI 0.81–0.93) and 0.89 (95% CI 0.80–0.94), respectively. For ^18^F-FDG-PET/CT in combination with contrast-enhanced CT, the pooled estimates for sensitivity were 0.95 (95% CI 0.88–0.98) and for specificity, 0.81 (95% CI 0.63–0.92) [221]. Similar data were reported by Gu A et al. [223]. To date, no results of prospective randomized studies are available, and the low incidence of resectable recurrencies will hinder the performance of prospective RCTs in the future. Indeed, none of the recruiting (NCT03398291, NCT04617457, NCT02892305, jRCTs051180199), or not-yet0recruiting (NCT05271110, NCT04975516) prospective RCTs reported before are exploring the treatment of oligometastatic metachronous PDAC. However, based on the so-far-available data, it is possible to define four useful criteria to identify patients who can benefit from the surgical removal of a resectable tumor recurrence: first, the chance of complete recurrence removal with R0 margins. second, an interval between surgical treatment of the primary tumor and recurrence of at least 9 to 10 months, since the longer the interval, the better the results; third, an assessment of the primary tumor response to neoadjuvant and/or adjuvant chemo/chemoradiotherapy to evaluate the opportunity for further chemo/chemoradiotherapy before reoperation; and fourth, an adequate evaluation of a tumor’s burden, tumor metabolic capacity, and the ability to metastasize as soon as available. Of course, a patient’s age and performance status should be considered.

In conclusion, the re-resection of a resectable PDAC relapse can give a significant survival benefit in carefully selected patients but further data are needed before it can be applied outside of high-volume centers with an interdisciplinary team of surgeons and oncologists.

## 5. Conclusions

The definition of marginally/borderline resectable PDAC was first introduced in the early 2000s [10,11]. While the definition of LA-PDAC is widely accepted, that of BR-PDAC has undergone repeated changes and remains under discussion. Currently, the most used guidelines to define BR- and LA-PDAC are those of the NCCN [22], and NAT represents the initial treatment of choice for both. A consensus statement on the best imaging procedures and their limitations to be performed before and after NAT in patients with BR-PDAC was reached by a group of experts [25].

However, the assumption that the anatomical characteristics used to define PDAC resectable, BR and LA, correspond to its well-defined behavior and that all patients who share the same anatomical profile must undergo the same sequence of treatments does not take into account the biological characteristics of PDAC [50,51,52]. Unfortunately, we are still waiting for an adequate definition of the role of different biomarkers in evaluating the prognosis of PDAC and its possible response to NAT, while there is not yet a generally accepted panel of immunohistochemical and/or biological biomarkers predictive of the evolution of the disease [53,54,55,56,57,58,69,70,71,73,74,75,76,77,78,79]. Furthermore, clinical decision making for patients with BR-PDAC may be altered by possible variability in the interpretation of CT images by radiologists and surgeons [34]. Resectability determined by post-NAT CT has a sensitivity and specificity of 95.2% and 8.7%, respectively [90], and this would limit the resection rate of resectable patients if not integrated with CA 19-9 assessment [59] or with positron emission tomography performed with ^18^F-FDG [102,114,119,120,121,122,123,124,125,126,128,130,132] or other tumor-specific tracers [138,139,140,147,148,149]. Several problems still exist: 1. how to select the most active chemotherapy regimen [81,82]; 2. how to predict early recurrence after radical resection [110,151,152]; and 3. which tumor-specific tracer can be used for PET/CT to provide a more accurate staging and better monitoring of therapeutic response.

Synchronous metastatic PDAC is generally considered unresectable by international guidelines [21,22,117,118,126], regardless of the size and number of distant metastases. Palliative systemic chemotherapy has been the standard of care for these patients. The term “oligometastatic” PDAC has recently been introduced, in which metastases are small in number and limited to a single or limited number of organs [156]. It was believed that similar to what had been achieved in similar conditions in colorectal, neuroendocrine, and gastric cancer, curative treatment might still be possible [157]. After several randomized trials demonstrating increased OS in patients with metastatic PDAC undergoing chemotherapy [190,191] and improved survival after surgical resection after NAT compared to after UFS [153,154,162,175,189], conversion surgery after a satisfactory response to NAT is considered the standard treatment of oligometastatic PDAC. However, high-level evidence demonstrating the benefit of conversion resective surgery after a satisfactory response to NAT is still lacking. There are six ongoing clinical trials on the treatment of oligometastatic PDAC. Until the results of these trials are available, the synchronous resection of PDAC and oligometastatic disease should only be performed within high-volume centers and in a research setting.

PDAC is an aggressive cancer, and almost all patients will develop disease recurrence after resection [196,197,198] even after R0 surgery [59,199]. The first report of a successful surgical treatment of locoregional recurrence without distant metastases was published in 1992 [201], but only in the 2000s did the first series from larger cohorts start to be reported [202,203]. The early diagnosis of metachronous PDAC metastases can bring the best therapeutic result and the highest chance for prolonged OS [210]. Tumor relapse is detected earlier by ^18^F-FDG-PET/CT than by CT [219,220]. Based on the available data, it is possible to define some useful criteria to identify patients who can benefit from the surgical removal of a tumor recurrence: first, the chance of complete recurrence removal with R0 margins; second, an interval between surgical treatment of the primary tumor and recurrence of at least 9 to 10 months; third, an assessment of the primary tumor response to NAT and/or adjuvant chemo/chemoradiotherapy to evaluate the opportunity for further chemo/chemoradiotherapy before reoperation; and fourth, an adequate evaluation of the tumor’s burden, tumor metabolic capacity, and the ability to metastasize as soon as possible. Of course, a patient’s age and performance status should be considered. Of particular interest is that the reported mortality rate in the case of re-resection is between 1 and 2% [216,217]. To date, no results of prospective randomized studies are available. In conclusion, the re-resection of a resectable PDAC recurrence can provide a significant survival benefit in carefully selected patients, but high-level evidence demonstrating that the outcome is due to surgical treatment and not to patient selection is still lacking. More data are needed before it can be applied outside of high-volume centers with interdisciplinary teams of surgeons and oncologists.

## Figures and Tables

**Table 1:** M. D. Anderson criteria for resectability of pancreatic cancer.

Vessel	Resectable	Borderline Resectable	Locally Advanced
SMA	No extension; normal fat plane between the tumor and the artery	Tumor abutment ≤ 180° (one half or less) of the circumference of the artery; periarterial stranding and tumor points of contact forming a convexity against the vessel improve chances of resection	Encased (>180°)
Celiac axis/ Hepatic artery	No extension	Short-segment encasement/abutment of the common hepatic artery (typically at the gastroduodenal origin); the surgeon should be prepared for vascular resection/interposition grafting	Encased and no technical option for reconstruction usually because of extension to the celiac axis/splenic/left gastric junction or the celiac origin
SMV/PV	Patent	Short-segment occlusion with suitable vessel above and below; segmental venous occlusion alone without SMA involvement is rare and should be apparent on CT images	Occluded and no technical option for reconstruction

SMA, superior mesenteric artery; SMV/PV, superior mesenteric vein/portal vein; CT, computed tomography. Reprinted from Varadhachary GR with permission [16].

**Table 2:** International consensus of classification of BR-PDAC based on anatomical definition using CT imaging, including coronal and sagittal sections.

**Resectable: R**	SMV/PV: no tumor contact or unilateral narrowing SMA, CA, CHA: no tumor contact
**Borderline resectable: BR**	Subclassified according to SMV/PV involvement alone or arterial invasion.
BR-PV (SMV/PV involvement alone)	SMV/PV: tumor contact 180° or greater or bilateral narrowing/occlusion, not exceeding the inferior border of the duodenum. SMA, CA, CHA: no tumor contact/invasion
BR-A (arterial involvement)	SMA, CA: tumor contact of less than 180° without showing deformity/stenosis. CHA: tumor contact without showing tumor contact of the PHA and/or CA. (The involvement of the aorta is categorized as unresectable. Presence of variant arterial anatomy is not taken into consideration)
**Unresectable: UR**	Subclassified according to the status of distant metastasis.
Locally advanced: LA	SMV/PV: bilateral narrowing/occlusion, exceeding the inferior border of the duodenum. SMA, CA: tumor contact/invasion of 180 or more degrees #. CHA: tumor contact/invasion showing tumor contact/invasion of the PHA and/or CA. AO: tumor contact or invasion
Metastatic: M	Distant metastasis $.

SMV: superior mesenteric vein, PV: portal vein, SMA: superior mesenteric artery, CA: celiac artery, CHA: common hepatic artery, PHA: proper hepatic artery, AO: aorta. #: In the cases with CA invasion of 180° or more without involvement of the aorta and with intact and uninvolved gastroduodenal artery, thereby permitting a distal pancreatectomy with en bloc celiac axis resection (DP-CAR), some members prefer these criteria to be in the BR-A category. $: including macroscopic para-aortic and extra abdominal lymph node metastasis. Reprinted from Isaji S with permission [23].

**Table 3:** Classification of BR-PDAC based on anatomical, biological, and clinical aspects.

Type of Definition	Anatomical		Biological	Conditional
**R**	R-Type A		No: R-Type A	No: R-Type A
			Yes: BR-Type A	Yes: BR-Type C
**BR**	BR-Type A		No: BR-Type A	No: BR-Type A
			Yes: BR-Type AB	Yes: BR-Type AC
**Locally advanced: LA**	LA-Type A		No: LA-Type A	No: LA-Type A
			Yes: LA-Type AB	Yes: LA-Type AC
**Biological definition:**		CA 19-9 more than 500 IU/mL Regional lymph node metastasis (biopsy or PET-CT)		
**Conditional host-related definition:**		Depressed performance status (PS: 2 or more)		

Tumor is classified based on combination of A, B, and C (for example, a patient with both Type-B and Type-C features would be classified as Type ABC). Grey Color highlights the definitions that characterize BR (borderline resectable PDAC). Reprinted from Isaji S with permission [23].

**Table 4:** Tumor response grading systems for PDAC resected post NAT.

TRG	Evans 1992		CAP 2005				MDACC 2012	HTRG 2016	RNS 2021	IPSMDA 2023	IPSCAP 2023
High	Grade 4 No viable tumor cells present.		Grade 0 No viable residual tumor				Grade 0 No residual carcinoma	HTRG 0 no residual carcinoma within entire pancreas, bile duct and ampulla of Vater	Grade 1 0–10% of tumor bed area occupied by viable carcinoma	IPSMDA 0–3 ‡	IPSCAP 0–3 ‡
	Grade 3 <10% viable-appearing tumor cells present		Grade 1 Single cells or small groups of cancer cells				Grade 1 Minimal residual carcinoma *	HTRG 1 minimal residual carcinoma †			
Intermediate	Grade 2B Destruction of 51–90% of tumor cells		Grade 2 Residual cancer with evidence of tumor regression, but more than single cells or rare groups of cancer cells				Grade 2 5% or more carcinoma in treated tumor bed	HTRG 2 5% or more residual carcinoma	Grade 2 11–75% of tumor bed area occupied by viable carcinoma	IPSMDA 4–5 ‡	IPSCAP 4–6 ‡
	Grade 2B Destruction of 10–50% of tumor cells										
Low	Grade 1 <10% or no tumor cell destruction		Grade 3 Extensive residual cancer with no evidence of tumor regression						Grade 3 76–100% of tumor bed area occupied by viable carcinoma	IPSMDA 6–7 ‡	IPSCAP 7–8 ‡
N. patients	223 ¥	147 #	223 ¥	147 #	167 ††	472 ^a^ ||	147 #	167 ††	147 #	398 ‡‡	398 ‡‡
High %	2.7–16.1	**4.1–6.8**	2.7–16.1	4.1–8.2	1.8–10.8	26.1	4.1–8.8	1.8–10.8	13.6	27.9	23.9
Intermediate %	55.6–17.5	15.0–44.9	55.6	56.4	56.9	**44.5**	87.1	87.4	72.8	54.3	67,6
Low %	**8.1**	29.2	25.6	31.3	30.5	29.4			13.6	17.8	8.5
										
SSDOSC	0.004	0.039	0.001	0.026	0.07	1.00 ^b^ 1.42 ^b^ 1.45 ^b^	0.021	0.02	<0.0001	<0.0001	<0.0001

TRG: Tumor response grading; ^a^: 581 minus 109 unknown as CAP score was not regularly documented before 2014; SSDOSC: Statistically significant difference of overall survival curves; ^b^: Overall survival HR (95% CI). The lowest and highest percentage values of each TRG are bold or underlined, respectively. *: Single cells or small groups of cancer cells; <5% residual carcinoma in treated tumor bed; †: Single cells or small groups of cancer cells, <5% residual carcinoma; ‡ The IPS was calculated as the sum of the scores for ypT (score 0, 1, 2, and 3 for ypT0, ypT1, ypT2, and ypT3, respectively), ypN (score 0, 1, and 2 for ypN0, ypN1, and ypN2, respectively), and TRG according to either MD Anderson grading system (IPSMDA) or CAP grading system (IPSCAP). The TRG scores for MDA system were 0, 1, and 2 for MDA grade 0, 1, and 2, respectively, and the TRG scores for CAP system were 0, 1, 2, and 3 for CAP grade 0, 1, 2, and 3, respectively. ¥: Chatterjee D [92]; #:Chou A [93]; ||: Habib JR [94]; ††: Lee SM [95]; ‡‡: Sohn AJ [96].

**Table 5:** Criteria for simultaneous resection of PDAC and liver metastases.

Authors	n.	Identified Criteria
Takeda et al., 2023 [160] (2013–2020)	12	CA19-9 normalization. Objective response to chemotherapy for at least 8 months.
Wu et al., 2023 [178] (2009–2017)	6	Resectable with a good anatomic setting.
Nagai et al., 2023 [153] (2000–2019)	47	Pathologically confirmed metastatic PDAC to the liver. Pathologically confirmed metastasis to an extrahepatic site identified at surgical exploration excluded.
Bachellier et al., 2022 [166] (2008–2020)	92	Contraindications: Persistent high CA19-9. Preoperative need for venous resection and/or the presence of venous invasion.
Frigerio et al., 2022 [161] (2008–2020)	52	Disappearance of liver metastases at cross-sectional imaging. Negative 18FDG-PET. Serum carbohydrate antigen (CA19-9) decrease threshold > 50% relative to baseline was employed to define biochemical response.
Hank et al., 2022 [189] (2006–2019)	67	Stable disease, partial, or complete response of the primary tumor and metastatic lesions based on the RECIST criteria. Biological tumor response (decrease in CA19-9 and carcinoembryonic antigen (CEA)). Technically resectable metastatic disease. Performed only at a high-volume centers.
Safi et al., 2021 [179] (2006–2019)	35	Oligometastatic disease was defined as resectable hepatic metastases isolated in one hepatic lobe, accessible only via an atypical resection and independent of size and amount of metastases.
Gu et al., 2020 [171] (2003–2014)	29	Possibility of resection. Patient and family consent.
Shao et al., 2020 [159] (2009–2018)	50	Liver oligometastases with good resectability. Good response to NAT (reduction of CA 19-9 more than 50%). Baseline CEA no more than 8 ng/mL. Primary PDAC with achievable R0 resection. Good performance status for surgery.
Yang et al., 2020 [180] (2012–2017)	48	No extrahepatic metastases. An R0 resection of the primary PDAC can be performed. The liver metastases can be completely extirpated by operation or operation combined with RFA. Patient’s general condition good, with ASA score < III. Venous resection or multivisceral resection accepted.
Andreou et al., 2018 [181] (1993–2015)	76	Liver resection was considered if safe removal of pancreatic liver metastases was possible and the liver remnant was deemed sufficient.
Shi et al., 2016 [182] (2007–2015)	30	Good performance status. Intention to reach a R0 status in both the pancreas and the liver.
Slotta et al., 2014 [183] (Six ear period)	9	Patients’ performance status. Resectability with adequate oncological resection margins and a maximal volume of functional hepatic parenchyma remnant. Declared patients’ will and informed consent. Non-resectability for infiltration of all three liver veins, diffuse liver Metastases, and non-resectable extrahepatic tumor manifestations.
Singh et al., 2010 [184] (2003–2009)	7	Synchronous liver resection was performed only if an R0 resection of the pancreatic tumor was possible.
Seelig et al., 2010 [185] (2004–2007)	20	The impression to reach an R0 situation with synchronous resection of metastasis. Good clinical performance status of ASA III or better. Patient’s will to receive maximal treatment.
Dünschede et al., 2010 [186] (1996–2008)	9	Possibility of R0 resection of the primary and/or the liver metastases. No other sites of metastases.
Yamada et al., 2006 [187] (1991–1995)	5	The feasibility of a complete excision of all intrahepatic disease. Reliable control of the primary disease by means of extirpation. No extrahepatic diseases at the time of detection of resectable liver metastases.
Takada et al., 1997 [188] (1981–1995)	11	Indications: Complete removal of the tumor with a histologically clear margin. Removal of the peripancreatic soft tissue and removal of metastasis from the primary and secondary lymph node groups. Contraindications Reasonable certainty that a resection of the primary carcinoma of the pancreatic head or the liver metastasis would not result in a cure. The patient would be unable to tolerate aggressive surgery.

RECIST: Response Evaluation Criteria in Solid Tumors; RFA: Radiofrequency ablation; ASA: American Society of Anesthesiologists.

**Table 6:** Treatment of metachronous metastases after pancreatic cancer resection.

Author	n.	SMMR	RMM	NRMM	M %	MOS †	DFI †	MPRS †	MSB †	CS †	DFSPR †
Serafini et al. [204]	6	ILR	176	255	1.1	Almost twice ‡ *p* = 0.006	NR	+15.2 *p* = 0.002	28.7 <0.001	NR	NR
Choi et al. [205]	15	ILR	50	0	2.0	NR	41.3 + 29.09 ¥	60 ¥	NR	107	NR
Guerra et al. [206]	15	IPuR	286	799	NR	34.7 vs. 7.3	15.9 ¥	16.5 ¥	NR	NR	NR
		ILR	708	NR	NR	23.6 ¥	12.3 ¥	9.7 ¥	NR	NR	NR
		ILiR	681	NR	NR	11.3 ¥	7.3 ¥	8.0 ¥	NR	NR	NR
		IPeR	173	NR	NR	17.9 ¥	8.5 ¥	5.5 ¥	NR	NR	NR
Liu et al. ‡ [207]	20	IPuR	147	190	NR	NR	16.0 to 52.5 vs. 10.5 to 22.0	4.0 to 44.0 vs. 8.1 to 31.3	NR	NR	NR
Moletta et al. [208]	14	ILR	230	NR	1.8	68.9 #	24.3 #	26.0 #	NR	NR	14.2
Sakaguchi et al. [209]	19	ILiR	343	NR	NR	24.5 to 40.0 ||	7.6 to 18.4 ||	11.4 to 31.0 ||	NR	NR	NR
		IPuM	57	NR	NR	51.0 to 85.9 ||	24.0 to 52.4 ||	18.6 to 38.3 ||	NR	NR	NR
		IPeM	28	NR	NR	5.3 to 12.9 ||	NR	NR	NR	NR	NR
Lovecek et al. [210]	17	IPuM	70?	180?	NR	NR	NR	NR	NR	NR	NR
Groot et al. [211]	8	ILR	100	5969	1	42.5 to 79.5 ¥	25.0 to 50.5 ¥	16.0 to 32.0 ¥	NR	NR	NR

†: months; ‡: RMM compared to NRMM; ¥: reported only range of RMM patients; #: reported only range of RMM patients due to the lack of single patient outcome in unresected group; ||: reported only range of RMM patients; statistical analysis or meta-analysis was not performed due to the high heterogeneity in reporting of outcomes among eligible studies. SMMR: site of metachronous metastasis resected; RMM: resected metachronous metastasis; NRMM: not-resected metachronous metastasis; M: mortality after RMM (%); MOS: median overall survival after pancreatic resection; DFI; disease-free interval between 1st surgery and re-resection or diagnosis of tumor relapse; MPRS: median post-recurrence survival; MSB: median survival benefit; CS: conditional survival months; DFSPR: disease-free survival after re-resection. IPeM: isolated peritoneal metastasis; ILR: isolated locoregional recurrence localized to the posterior resection margin, the pancreatic remnant, or the locoregional lymph nodes; IPuR: isolated pulmonary recurrence; ILiR: isolated liver recurrence; IPeR: isolated peritoneal recurrence.

## Abbreviations

AHPBA: American Hepato-Pancreato-Biliary Association; AIOM: Italian Association of Medical Oncology; BR-PDAC: borderline resectable pancreatic ductal adenocarcinoma; CAFs: cancer-associated fibroblasts; CMR: complete metabolic response; CTC: circulating tumor cells; DOPs: patient-derived organoids; ECs: endothelial cells; ESMO: Society for Medical Oncology; FAPI: Fibroblast Activating Protein Inhibitor; ^18^F-FLT: ^18^F-Fluorothymidine; HA: hepatic artery; IAP: International Association of Pancreatology; ISGPS: International Study Group on Pancreatic Surgery; LA-PDAC: locally advanced pancreatic ductal adenocarcinoma; LN: lymph node; MDCT multidetector computed tomography; MRI: magnetic resonance imaging; NAT: neoadjuvant therapy; NACRT: neoadjuvant chemoradiotherapy; NCCN: National Comprehensive Cancer Network; UK NIHCE: UK National Institute for Health and Care Excellence; OS: overall survival; PET/CT: positron emission tomography computed tomography; PDAC: pancreatic ductal adenocarcinoma; PSMA: prostate-specific membrane antigen; RCTs: randomized controlled trials; RM: resection margin; SMA: superior mesenteric artery; SMV-PV: superior mesenteric vein-portal vein; SSAT: Society for Surgery of the Alimentary Tract; SSO: Society of Surgical Oncology; SUV: standardized uptake value; UFS: upfront surgery.
